# Telling Moments and Everyday Experience: Multiple Methods Research on Couple Relationships and Personal Lives

**DOI:** 10.1177/0038038515578993

**Published:** 2015-10

**Authors:** Jacqui Gabb, Janet Fink

**Affiliations:** The Open University, UK; University of Huddersfield, UK

**Keywords:** couple relationships, everyday experience, mixed methods, moments approach, relationship practices

## Abstract

Everyday moments and ordinary gestures create the texture of long-term couple relationships. In this article we demonstrate how, by refining our research tools and conceptual imagination, we can better understand these vibrant and visceral relationships. The ‘moments approach’ that we propose provides a lens through which to focus in on couples’ everyday experiences, to gain insight on processes, meanings and cross-cutting analytical themes whilst ensuring that feelings and emotionality remain firmly attached. Calling attention to everyday relationship practices, we draw on empirical research to illustrate and advance our conceptual and methodological argument. The *Enduring Love?* study included an online survey (*n* = 5445) and multi-sensory qualitative research with couples (*n* = 50) to interrogate how they experience, understand and sustain their long-term relationships.

## Introduction

Everyday life is a life lived on the level of surging affects, impacts suffered or barely avoided. It takes everything we have. But it also spawns a series of somethings dreamed up in the course of the things. (Stewart, 2007: 9)

In her book *Ordinary Affects*, Kathleen Stewart endeavours to slow down the pace of analytical thinking as a means of speaking to and taking account of complex and uncertain objects, ‘to fashion some form of address that is adequate to their form; to find something to say about ordinary affects by performing some of the intensity and texture that makes them habitable and animate’ (2007: 4). In so doing Stewart resists analytical closure and seeks to retain the ‘messiness’ (Daly, 2003; Gabb, 2009, 2011) or, in her terms, the complexity and uncertainty which comprise ordinary ‘things’ (Stewart, 2007: 5). In this article we advocate a similar analytical strategy for the study of long-term couple relationships, focusing attention on the incidental, the often unnoticed and the ephemeral which create the texture of such relationships and through which their tensile strength is constituted.

Relationships comprise pragmatics and emotions, choices and lack of choice, contentment and disenchantment – and all the spectrum of feelings and experiences in-between. Research has added significant insight into the range of affective attachments that comprise intimate life (Duncan and Phillips, 2008; Heaphy et al., 2013; Jamieson et al., 2006; Roseneil, 2005; Smart, 2007), but there remains a particular absence of sociologically-informed studies of *couples in long-term relationships*, with regard to both the influence of culture, biography and socio-economic factors on their relationship experience and the interiority of their personal lives (Smart, 2007). This is a significant gap not least because couple relationships in contemporary Britain have continuing appeal across the sexual spectrum despite shifts in the configuration of intimacy and intimate living (Giddens, 1991; Jamieson, 1998). Married couples, for example, still head up seven in ten households in Britain, with rates of marriage increasing by 5.3% between 2011 and 2012 (ONS, 2014), and 46,000 same-sex partnerships being registered between December 2005 and 2010 (ONS, 2011).

Through the *Enduring Love?* study,^1^ we have sought, therefore, to address this under-researched dimension of heterosexual and non-heterosexual relationships while also situating emotions at the conceptual, methodological and analytical heart of our inquiry. In this we acknowledge the queer critique that has been rallied against ‘the couple’ and coupledom (Wilkinson, 2013) but argue for more nuanced approaches to the conceptualization of the couple because of the wide diversity in their lived lives and the different ways in which couples give meaning to and sustain their relationships together over time (see Gabb and Fink, 2015). More specifically in this article we focus on how everyday experience in long-term relationships makes and remakes couple intimacies in dynamic and emotionally charged configurations.

To achieve this we deployed a multiple methods, multi-sensory research design to access accounts of vibrant and visceral relationships, foregrounding the *everyday* and focusing on *ordinary moments* as a lens through which to examine relationship process, practice and structure. This ‘moments approach’ is, we argue, a dynamic means to advance understandings of patterns of relationship experience, grounded in their biographical contexts and emotional settings. It affords us a way of staying attached to the ‘everydayness’ of relationships (Daly, 2003), providing close-up insights that effectively and affectively capture the essence of relationships. This article illustrates, then, how a moments approach to studying couple relationships and intimate life more generally can open up an analytic crack through which we can better see and shed light on personal experience and thereby facilitate multidimensional analysis of the materiality, temporality and emotionality of everyday lives.

To begin, we introduce how the everyday has been deployed in studies of family and intimate life as a means to interrogate and make sense of personal relationships. We situate our research in the context of the ‘practices approach’ which has been so influential in UK family sociology and in the rich tradition of methodological creativity that increasingly characterizes this field and emotional geographies more widely. The *Enduring Love?* study is used to inform the conceptual argument of the article and provide methodological illustration. We elucidate how and why we analysed the quantitative and qualitative datasets from the project, paying particular attention to the value and meanings of those everyday ordinary moments that were our analytic foci. We argue that such moments shed light on the different patterning of relationship experience we identified in the project’s datasets, including how sex and love, adversities and ambivalences, care and support and the public/private boundary are managed as part of the everyday relationship work that couples do. Our focus on the qualitative data from two couples and two small everyday moments in their relationships is thus intended to exemplify the way a moments approach reveals such patterning and can provoke wider concurrent thematic analysis across each couple’s dataset and the project’s dataset as a whole. In conclusion, the article acknowledges the costs and benefits in using everyday moments to study personal life. The approach is resource intensive, but starting with minutiae and mundanities productively reorients the analytical lens onto the *vitality* of relationships, as they are lived in both time and space.

## Everyday Lives and Emotion

Our moments approach has at its conceptual and epistemological heart the belief that couple relationships are constituted, experienced and afforded meaning through the everyday. It is informed by an understanding that emotions are embedded in social relations and that moments can be identified as ‘emotional scenarios’, in which micro and macro networks of relations intersect and overlap (Burkitt, 2014: 20). It aims to keep the constitutive and iterative process of *doing* relationships at the forefront of analysis (Morgan, 1996) while calling attention to the interdependent elements that last beyond specific moments of enactment (Phoenix and Brannen, 2013). It also acknowledges the extent to which everyday life informs the conduct and conceptualization of much family research and study of intimate life (Gabb, 2008). In this context, however, everyday life is not simply viewed as a process through which habits (or in Bourdieusian terms *habitus*) impel the self-governing individual to conform. Routines and practices are vibrant and visceral (Martens et al., 2013) and can be a site of coherence and contestation. Routinization may render invisible the processes and structures through which relationships are understood and afforded meaning, but through iteration and the diurnal what was once different may, over time, become ‘normal’: the marginalized can become mainstream (Heaphy et al., 2013). This temporal dimension of how relationships are made and re-made is crucial: the quotidian of life is dynamic. Experience is embodied and located in the specificity of place (Pink, 2012). Everyday practices are configured and reconfigured over time (Shove et al., 2012). Relationships also involve investments that weave together a shared past, the present day and imagined futures, which, in turn, illustrate a diachronic dimension to the everyday. We situate our argument, therefore, within this conceptual framework of the everyday, while at the epistemological core of our proposition is the contention that we need to refine our research tools and conceptual imagination to appreciate the meaningfulness of everyday ordinary moments in personal life.

This attention to *the momentary* draws on and extends ideas developed in social theorizing. Notably, Anthony Giddens (1991) has characterized ‘fateful moments’ as being a central part of late modernity, playing a significant and formative role in the emergence of individualization. Giddens defines fateful moments as critical turning points when the life of an individual (or collective) shifts (1991: 112–13). Relevant examples here of such ‘crossroads’ would include the decision to get married and/or start a family. Fateful moments are thus associated with key life events and the epiphanal. In family studies there has been some attention to moments as a way to understand personal life. However, perhaps driven by a Giddensian agenda, the focus has tended to be upon *extraordinary* or special moments (Larson and Bradney, 1988) or specific dimensions of family life, interrogating phenomenon such as ‘quality time’ (Kremer-Sadlik and Paugh, 2007). Notwithstanding the insights provided through these foci, the momentary concept has, however, been problematized through empirical research, being critiqued for effacing power, and the problematic and provisionality of lived lives (Plumridge and Thomson, 2003).

More broadly the momentary has been explored in anthropological contexts through the idea of a ‘revelatory moment’ and its value as an ‘epistemic unit’ through which researcher subjectivity and socio-cultural significance can be examined (Trigger et al., 2012). Autobiographical studies and anecdotal theorizing (Gallop, 2002) use incidental moments to build theoretical understanding from the bottom up, drawing upon the personal as a lens through which new perspectives on relationality can be brought into view (Gabb, 2011). Psycho-social research has explored everyday ‘encounters’ which tip us off balance; ‘moments of undoing’ which have the capacity to make us stop short and think again, perhaps from a new point of departure (Baraitser, 2009: 3). In all these instances moments are used to unsettle simple readings, situating the subject of inquiry, the participant and researcher in biographical and socio-cultural contexts that inform the research process and data generated. We draw upon these different conceptualizations of the momentary but our approach also understands the moment as having an emotional scenario at its core, as we noted above, and moments as where couples *feel* the immediacy of their intimate connections with each other (Burkitt, 2014). Our aim, then, is to demonstrate the value of the everyday ordinary moment as an analytical lens through which to advance understanding of relationship experience and practice.

## The *Enduring Love?* Study

The *Enduring Love?* project was a large-scale multiple methods study. A quantitative survey was designed to generate statistical information on relationship qualities, relationship with partner and relationship maintenance, enabling us to scope trends in behaviour and the factors which signal relationship satisfaction. The survey, primarily implemented online, was completed by 5445 people, including a UK convenience sample of 4494. Because we want to situate data in their particular personal-social contexts, our analysis is focused on the data from the UK cohort only.

The sample for the subsequent qualitative study comprised 50 couples, aged between 18 and 65 years. Of these couples, 70% were heterosexual and 30% lesbian, gay, bisexual and queer (LGBQ), 50% were parents and 50% were couples without children. Our selection of qualitative methods, emotion maps, diaries, individual interviews and photo-elicitation interviews with couples, was informed by a determination to drill down into realms of embodied lived experience. Emotion maps and diaries were completed simultaneously over the course of one week, with the former designed to locate everyday interactions in the home and depict the emotional dynamic of the couple’s relationship (Gabb, 2009) and the latter to generate temporal data on daily routines and more immediately engaged accounts of everyday life. Individual interviews were focused on how relationships work, examining participants’ experiences and relationships across the life course before moving into explorations of their diaries and emotion maps. A series of collages, designed by the research team, were used in the couple interviews and invited participants to reflect on the project’s central research themes: relationship work, physical affection and sex, children and childhoods, money, ‘significant others’, social policy and welfare, and media representations of couple relationships. The complexity of our research design meant that fieldwork with each couple was generally completed over a one to three-month period and, for the majority, took place in the privacy of their home.

## Survey Data and Everyday Relationship Practices

The survey’s design and our analytical strategy drew on the work of others in the quantitative and mixed methods field (Browne et al., 2013), including psychological research which often deploys quantitative surveys including psychometric scales to advance understandings of relationship satisfaction (for an overview see Hook et al., 2003) and how people understand their couple relationships (Duck, 2007; Mashek and Aron, 2004). For example, in relationship studies a key psychometric scale is the Golombok Rust Inventory of Marital State (GRIMS) scale (Rust et al., 1986, 1990). Although the GRIMS scale productively informed our thinking and framing of the *Enduring Love?* survey statements, we ultimately discounted reusing it, electing instead to ‘go it alone’ and use independent statements that were more attuned to the project’s foci. We situate our analysis and this article, therefore, as part of the growing canon of methodological writing that focuses on the flexibility and fluidity of methods (Hesse-Biber, 2010) and celebrates paradigm and methodological diversity (Denzin et al., 2008). While debate continues on how to resolve the ‘incompatibility’ of underlying paradigms that characterize quantitative and qualitative methods (e.g. Cresswell and Clark, 2007; Guba and Lincoln, 1994), we have completed our analysis by simultaneously taking account of the epistemological and methodological issues that are at play when bringing together multiple methods data.

Our survey included three sets of multiple choice statements, using Likert scale responses ranging from 1 to 5. These spoke to the structuring interests of the *Enduring Love?* study overall and enabled us to scope trends in behaviour and the factors which appear to signal relationship satisfaction through responses to individual questions on relationship practice. The statistical survey was, therefore, designed to generate *vital* information on the patterning of relationship experience across a large and diverse convenience sample. We computed basic descriptive statistics for demographic information and some relationship outcomes. We also computed Multivariate Analyses of Variance (MANOVA) and correlation analyses to address more complex research questions including continuous and categorical variables. In advancing a ‘cluster analysis’ of relationship practices we have ultimately been able both to retain our conceptual focus and to use this quantitative dimension of the study to establish questions whereby to interrogate the smaller-scale qualitative dataset.

Using free-text open questions in the survey, participants were asked to tell us what they liked and disliked about their relationship and what their partner did that made them feel appreciated. These questions generated over 10,000 responses with answers ranging from several words to lengthy descriptions. The process of quantifying these qualitative data was a daunting task but they were all ultimately coded using grounded theory. In this way we organized the many emerging ideas and themes into clusters of answers; these informed the extended coding frame being developed for use with our qualitative data. As a consequence of this labour-intensive and time-consuming process, we are now able to complete mixed methods analysis across the study’s quantitative and qualitative datasets. Here, however, we will focus on the responses to just one of the free-text open questions: ‘Identify two things that your partner does for you that make you feel appreciated’. We use these data both to interrogate the patterning of relationship experience and to demonstrate why a quantitative survey can be a valuable instrument in studying everyday practices. For example, through our analyses of the data we move beyond satisfaction ratings and/or statistical prevalence of behaviour to reveal the ordinariness of things that are seen as relationship practices and the factors which count in shaping couples’ experiences.

**Figure 1.**
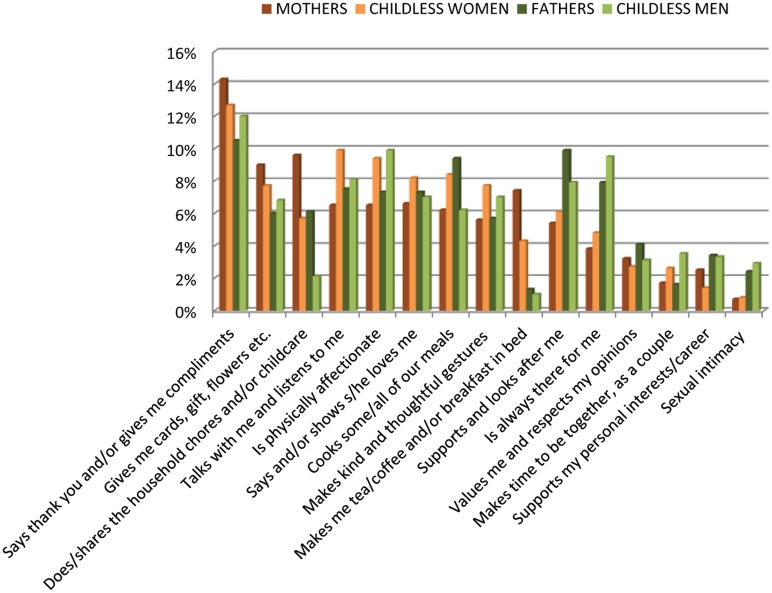
Identify two things that your partner does for you that make you feel appreciated.

Survey responses to the query ‘Identify two things that your partner does for you that make you feel appreciated’ illustrate the range of ‘things’ that were included by respondents, ranging from verbal expressions of gratitude to sexual intimacy; the latter of which being perhaps the most unexpected dimension to feature as an expression of appreciation. Here we draw attention to the prevalence, meanings and importance afforded to everyday relationship practices. Surprise gifts, thoughtful gestures and the kindness of a cup of tea in bed were all valued highly. Disaggregating these into separate categories (see Figure 1) reveals the nature and practices of the gestures being referred to. When combined, however, they comprise the most popular category for women, with 22% of mothers and 20% of childless women ranking everyday attentive acts as one of their two things which make them feel appreciated. Typical sentiments expressed in these responses included:

Warms my car up in the morning.

Picking a bunch of wild flowers and putting them in a vase for me.

Quantitative data from the *Enduring Love?* study indicate that the ‘success’ of a relationship for our participants was not dependent on money, the external validation afforded through socio-cultural external markers such as extravagant bouquets of flowers which profess to display love, or the approbation of extended kin. It was intimate *couple knowledge*, and its manifestation through everyday acts, that counted:

Gives me time to myself when he knows I’m jaded.

Shares private smiles when we are out.

Domestic roles and responsibilities rested alongside a sense of commitment and togetherness. Relationship practices and relationship generosity were meaningful because they demonstrated how both the relationship and the other partner were cherished. There is, then, an acknowledgement and valuing of the everyday practices and emotions that go into sustaining relationships over time (Gabb et al., 2013). Such endeavours were appreciated as relationship ‘gifts’ (Hochschild, 2003); acts of reciprocity that bound the couple together through give and take.

The characterization of and importance afforded to everyday gestures in the survey, and to the way these gestures were recurring features in often brief interactions between couples, thus ensured our sensitivity and attentiveness to the momentary as a defining quality of relationship practices. In the qualitative dataset, through our multi-sensory research design, we began therefore to more fully explore the *significance of momentariness*: how moments both shape everyday embodied lives and facilitate analytical explor-ation of the ways in which participants’ emotional–social worlds intersect.

## Multiple Methods in Multi-sensory Qualitative Research Design

While interviews often remain the default qualitative technique, creative qualitative methods have been developed and widely deployed in research with children and/or cross-generational and family studies (for discussion, see Gabb, 2008). For example in participatory research with children and young people, visual methodologies are frequently used to explore ideas of childhood which extend beyond the range and scope of the purely logocentric, and through which participants can narrate their own experiences and perspectives (Holland et al., 2010; Lomax et al., 2011). Visual methods are also drawn upon to extend understandings of the ways in which social inequalities shape the everyday experiences of communities, families and individuals (Fink and Lomax, 2012). In therapeutic contexts, systemic psychotherapists use a variety of nonverbal methods in their work with children and families (Wilson, 2007) including participatory action methods (Chimera, 2013). The graphic emotion map technique pioneered by Gabb in her research on family relationships (Gabb, 2008) has now also been extended for use in clinical contexts (Gabb and Singh, 2014a, 2014b). The evident usefulness of visual and embodied qualitative methods in these different research, participatory and therapeutic contexts reinforces our argument, therefore, that a qualitative multiple methods approach is vital in research on couple relationships and personal lives.

More particularly, through a multi-sensory multiple methods research design, richly textured data are generated which can focus on different dimensions of phenomena (Gabb, 2008). This layering approach has been variously described as pieces of a jigsaw (Gabb, 2009) or fragments of data which produce meaning through ‘each twist of the analytical kaleidoscope’ (McCarthy et al., 2003: 19). Mason (2011) has argued that facets (data from different methods) cast and refract light to afford ‘flashes of insight’ on a phenomenon (the multi-faceted gemstone). For us, however, there is no single gemstone. Relationships are highly complex phenomena in that the object of study is often hard to grasp, not least because it includes the individual, the couple, the social unit, as well as the biographical contexts of their lives and loves, and so on and so forth. The analytical foci can be difficult to sustain and, by default, are constantly evolving. Our approach, therefore, uses different methods to bring into sharp relief the range of everyday ordinary moments that, when combined, build up dynamic and multidimensional understandings of the couple relationship. Strands of data within these moments maintain individual integrity but they do not weave a seamless picture. It is the subtle interplay of cross-cutting threads that brings to the surface and works to retain the *emotional complexity* of lives lived in and through the everyday and in the context of wider social relations.

To analyse the qualitative data we deployed a process of thematic coding which has its roots in grounded theory (Charmaz, 2005; Miles and Huberman, 1983). Through reading and re-reading materials, structuring theoretical and experiential themes were identified and these were organized into clusters which resulted in the development of a 27-item coding frame. This thematic frame was then further broken down to identify different dimensions within these broad clusters. For example within the ‘tree node’ childcare there were ‘free nodes’ for child/family-centred, absence/presence of children, age/maturity and family planning.^2^ Given the sheer volume and complexity of data, the design and implementation of the coding frame was extremely time consuming. The reason that we invested so heavily in this labour-intensive process was to gain familiarity with the data, to ensure that the coding frame captured the richness and breadth of the data, and so we could systematically analyse the data through different analytical vectors – as couple cases, individual accounts, and via cross-sectional themes and attributes.

It is commonplace when using data management software to include a ‘gold dust’ code through which ‘juicy’ quotes can be identified. These are often used to lead early analysis. Our reticence at having this free-floating code was that when faced with an overwhelming volume of data it can serve to close down analysis, providing a shortcut to findings that obscures the complexity of the dataset. Gold dust can all too easily become sound bites which illustrate headline-grabbing claims, undermining otherwise nuanced multidimensional close analysis. We wanted both to avoid these analytical traps and to retain our focus on the everyday and momentary that had been so crucially highlighted in the project’s survey data. After all thematic coding had been completed we therefore worked into the coded dataset again to identify moments. These moments ranged from one line to several pages in length and featured in different forms in data from all the different methods.

Recognizing what constitutes ‘a moment’ was considered at length by the research team. It could be an apparently inconsequential activity in the course of an average day, a particular interaction between the couple or a response to an outside stimulus such as a TV programme. Whereas Giddens (1991) identifies the epiphanal as the identifiable mark of the fateful moment, our ‘revelatory moments’ (Trigger et al., 2012) were recognized by the researcher because they were *intimately revealing*. They were emotional scenarios which revealed something about the ways in which couples related to and interacted with each other, while also showing how those dynamics intersected with wider social relations, such as masculinity and femininity (Burkitt, 2014). In the remainder of this article, therefore, we concentrate on two such moments, drawing on the data from two couples: Sumaira and her partner and Hayley and her partner. By paying attention to the particularities of storytelling and visual tropes deployed in Sumaira’s diary and the tensions and ambivalences in Hayley’s diary we can see how these methodological and epistemological features combine to portray particular kinds of relationship stories. In so doing we advance understanding of both these couple relationships and the ways in which biography, experience and socio-cultural contexts intersect, thereby exemplifying the value of a moments approach in the analysis of qualitative methods data.

## What a Feeling: Adventures and Adversities

Diaries are a particularly useful method in studying personal relationships because they serve, in some ways, as a ‘confessional’ device that can shed light on and/or measure dimensions of private life (Harvey, 2011). They provide insight into couples’ everyday routines and the internal ‘couple lexes’ (Gabb, 2008). In the *Enduring Love?* study participants produced either hand-written or electronic format diaries and could also include visual mementos from the week’s events. For those with limited literacy skills diaries were completed retrospectively, in dialogue with the researcher, in verbal format. The length and format of completed diaries ranged from a few brief notes to lengthy and beautifully illustrated documents, an example of which we explore here:

I made dinner and [partner] came home. It was lovely to see him. We had a hug and chatted about our day. He got changed then we ate at the table together and I loved it so much. It’s perfect – just us and food. What more could I want? After dinner [partner] put a song on he likes and we danced which was funny. Then we went to [local area] for a walk. It was beautiful. We got lost and walked through grass which was taller than me and I got scared that it might get dark. We found our way through – just took a while! It was really nice – certainly an adventure. I would go there again. (Sumaira, diary extract)

**Figure 2.**
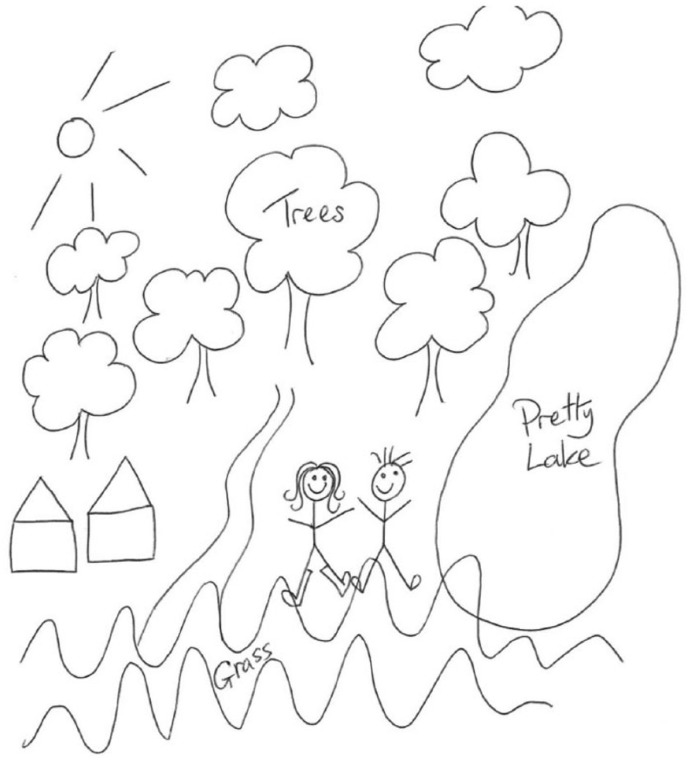
Sumaira’s diary. I drew this while I wait for [partner] to finish. It is where we went for our walk. I got to wear my wellies! That’s when you know it’s an adventure!

In this one diary extract and in the visual illustration (see Figure 2), Sumaira describes a single moment in her day. What it tells us about her and her couple relationship is, however, immeasurable and it serves to initiate broader direct and indirect analysis of our research themes, in particular generation, parenthood and gender. Throughout her diary Sumaira adopts the position of storyteller, a role that she appears to relish and through which she recounts her past, present and imagined future. Through an everyday homecoming scene she describes a traditional gendered norm of coupledom in which she depicts herself as the homemaker and her partner as the capable breadwinner. The dinner cooked for his return and its consumption at the dinner table discursively represent how things should be done, including the provision of quality couple time: ‘perfect’.

Notwithstanding the evident pleasure that was experienced in this dinnertime activity, there is a palpable sense of the satisfaction that Sumaira experiences in writing its description. This diary is evidently written with the reader in mind: the perfect time that Sumaira crafts also appears to be framed for *our* benefit. This young couple, both in their 20s and in full-time employment, are starting out on their life together. They have recently bought their first home and are, as such, still establishing their everyday relationship practices. They are enacting what they perceive to be ‘married’ life, embodying the ‘couple norm’ and situating their home as a safe haven (Sibley, 1995). The ‘adventure’ that Sumaira describes stands, therefore, as a metaphor for the real life adventure of their imagined future together; a future in which they are heavily invested. However, throughout the extract she moves between first person singular ‘I’ and the plural ‘we’ of being a couple. This switch in standpoint is revealing. For example, shared activities are typically embodied: ‘we had a hug’, ‘we danced’, ‘we found our way through’. In contrast, her first person singular statements are a mixture of emotions including certainties, fears and anxieties: ‘I loved it so much’, ‘I got scared’. The picture that thus emerges has leaky edges and hints towards the frailty of the perfection. It evinces the work that is being asked of the visual–textual diary data in shoring up their life together.

A photograph also accompanies the diary entry, a copy of which was included in both parties’ data. The image depicts Sumaira walking in front of the camera (which her partner is holding), cutting a path through the middle of the long grass; striding out, proudly and confidently in her ‘wellies’. It is a beautiful image. Sumaira’s partner now displays it, stored and digitally enhanced through *Instagram*, as the background on his phone. As a signifier of their relationship, it represents not only the pleasure of the moment but also the unfurling of their life adventure together. Western culture has become saturated with visual imagery which often serves to represent and reproduce dominant ideologies (Rose, 2000). ‘Reading’ these encoded texts requires visual literacy and participants are often highly skilled and ‘knowing consumers’ in this regard. Two-dimensional ‘still life’ becomes something vital, and in the process of translation: ‘our reactions to them render them meaningful’ (Gabb, 2013: 640–41). Readings, and what the viewer brings to the encounter, thus complete the story. The tale recounted here and its visual portrayals have a childlike quality that draws on the trope of fairy tales. The scene is reminiscent of forays into the woods taken by the Grimm Brothers’ Hansel and Gretel and Little Red Riding Hood. The picture-perfect walk, complete with sunny skies and a ‘pretty lake’, morphs into a scary scene with grass as tall as the young woman: disorientation, getting lost and encroaching darkness ensue. An ‘adventure’ indeed, including the heroic rescue by a young man who finds them a way out of the wilderness and saves the day.

Our unpicking of this moment and the analysis we advance here is not disparaging. It neither seeks to belittle Sumaira nor her description of the scene. What we are drawing attention to are the socio-cultural resources that are deployed to recount the experience; resources upon which we also draw to enrich our understanding of the relationship and which also focus analytical attention onto the distinctiveness of insights gained from different methods. These require us to incorporate the different traditions and paradigms which shape different modes of enquiry into the body of our analysis. The extract of data and the momentary scene described are richly textured and steeped in epistemological and ontological meanings. These data are further nested in Sumaira’s individual data, in the couple data, and intersect with her partner’s data. As such its portrayal is a portal into the project’s questions about the meanings and experience of long-term relationships. Sumaira’s diarized account and pictorial representation encapsulate where her relationship with her partner is, at this point in time. She knowingly invests in and cherishes the positive things they have – closeness, fun, resilience. However, speaking to her diary and emotion map data in her individual interview, Sumaira also recounts the tough times that the couple have faced, together and apart, including the divorce and remarriage of her parents when she was a young child, extended-kin obligations, money worries and anxieties around settling down. She talks about the ‘depression’ of her partner and his positive strategies to manage this, including several references to listening to music and dancing along. She is, then, painting a dynamic picture of relationship process; the couple are actively working on their relationship having survived previous ups and downs. Their walking adventure reflects not only their lived experience and emotional journey through the legacy of their pasts, their present and planned future together but also their shared commitment to keeping each other, and their relationship, safe.

## Spilling From the Page: Ambivalence and Everyday Intimacy

As we have detailed above, participant diaries were designed to encourage reflection upon and description of everyday relationship experiences, offering participants the opportunity to organize their thoughts, confess fears and anxieties, and construct temporally-nuanced accounts of their couple relationship. Some were explicitly reflexive about this, indicating how they selected and edited their entries, but others shared very intimate aspects of their lives. The example from Hayley’s diary below illustrates one such intimate scenario and exemplifies how a moments approach allowed us to tease open the complicated dynamics of this couple’s intimate relationship while also being attentive to their relational biographies and the socio-cultural contexts of couple relationships more generally:

Got woken at 5.15 with [partner’s] phone … irritating as I feel knackered. Both of us begin to stir and as we always do, we begin to caress each other. This relaxes me so that I can sleep again however it has the opposite effect on him! We make love. Nice in one way, feel very close and intimate. Annoying in another as I could have had another hour’s sleep! Very selfish of me!

I don’t want to hurt his feelings so would never say this to him, and actually it is lovely to be intimate. (Hayley, diary extract)

Hayley’s desire for sleep over ‘making love’ at this point in the week reflects other daily entries in her diary, wherein she emphasizes the escalating demands of her work and her struggles with the early mornings and late nights needed to meet her commitments. In a few short lines, she uses the diary to express her conflicted feelings about this early morning disruption of her sleep. Opposites abound and spin out from the core of her account. She is irritated and relaxed; feeling nice and being annoyed; wanting sleep and enjoying sex; acknowledging her selfishness and denying its legitimacy. The visceral account of her tiredness and irritation is thus irreconcilably juxtaposed with the almost romanticized description of how ‘lovely’ it is to be intimate with her partner. The emotional intensity of this particular entry was very different from others in the diary and was, therefore, identified as a revelatory moment, both for Hayley and the research team’s understanding of potential tensions in her relationship. Picking up how Hayley actively works on her emotions in recording this moment in her diary thus drew analytic attention to her ambivalence more generally around the intimacy she shares with her partner and how it could be traced in her data as a whole.

Hayley’s emotion map, for example, provides a methodologically compelling portrayal of her stark differentiation between the many ‘happy’ instances of emotional and physical affection that she experiences with her partner in the garden and on the ground floor of their home and their seemingly minimal intimate encounters upstairs. This is reinforced in her individual interview where she describes further how she and partner interact, emphasizing the platonic but pleasurable nature of their affectionate relationship downstairs but that they ‘don’t really spend any time upstairs’. Hayley’s attempts to contain the sexual dimension of her relationship and her ambivalent feelings about it are evident, too, in her couple interview where she frames her responses to the collage about physical affection and sex as questions to her partner. Through these pointed invitations to speak, Hayley’s partner is encouraged to agree with her interpretation of their relationship and to offer reassurances that he is content with their life together. Such reassurances were immensely important to Hayley, as their couple relationship was a relatively short one. They had met and married in quick succession and were actively engaged in relationship work because, as they explained, both had experienced ‘failed’ marriages and were committed to ensuring the ‘success’ of this relationship.

What can be ‘heard’ (Back, 2007), then, in and through this moment is Hayley’s expectation that she should meet her partner’s need and desire for sexual intimacy as well as her understanding that it would be selfish to presume her needs might come before his. Even to articulate her needs risks hurting his feelings and damaging their relationship. She thus works to reassure herself and the researcher, as the ‘audience’ of her different narratives (Brannen, 2013), that the sexual intimacy she shares with her partner is mutually satisfying. In one respect the work she undertakes can be explained through the way women’s and men’s sexuality is gendered in socio-cultural discourses on love, intimacy and commitment and in perceived ‘norms’ that men are more sexually active than women. However, our moments approach also shows how her anxiety about this norm and the ambivalence she experiences combine to close down opportunities for her to imagine, explore or establish alternative ways of being together intimately with her partner. It elucidates both the differently gendered patterns of sexual experience in couple relationships and the points where gendered experience, expectation and socio-cultural norms coalesce in intractable ways. For Hayley, and despite the immense pleasure and contentment she experiences with her partner, there is no easy resolution of the moment that we have explored here or its potential to haunt her couple relationship over the longer term.

## Everyday Moments: The Costs and Benefits of Multiple Methods Research

A multiple methods moments approach is time intensive and resource heavy at all stages of the research process. The mass of data that is produced creates an unwieldy dataset that is hard to pin down and can feel overwhelming. The sheer volume of material can serve to obscure the object of analysis, that is to say, you cannot see the (analytical) wood for the (multifariously coded) trees. The particularities of individual biographies seem resistant to thematic analysis: everyone and each couple is different. The different methods and techniques deployed draw on different paradigms, requiring a broad range of analytical skills and producing frameworks of understandings that cannot be easily merged or transferred. Factoring distinctive paradigms into analysis can make it hard to read across the dataset. Our analysis did have multiple false starts and progress was at times faltering. Paying attention to the particular tropes, theoretical traditions and different qualities of data requires close and time-intensive multiple readings. The object of study in itself is dynamic and constantly shifting. Yet notwithstanding these practical challenges, analysing our rich data through a moments approach has allowed us both to understand experience and to appreciate and *feel* participants’ everyday lives. It has en-abled us to locate emotion at the centre of the analytical equation; something that is arguably invaluable in research on personal relationships. We have also, to return to the opening reference to Stewart (2007: 5), become more adept at interrogating the complexity and uncertainty which comprise ordinary ‘things’.

The success of the approach undoubtedly requires considerable researcher skill and imagination, and no small degree of confidence and determination. Its rigour is achieved through the iterative analytical process, grounded in the epistemological and methodological paradigms that remain attached to different methods. In the end, however, a leap of faith in the scientific imagination will inevitably be required. Notwithstanding the breadth, depth and scale of findings, focusing on moments of everyday experience is unlikely to provide definitive answers.

Nevertheless the moments included here have demonstrated the efficacy of the approach in opening up everyday meanings, experiences and understandings of couple relationships. The analysis has drawn attention to the specificity of the methodological medium and its aetiology. The use of quantitative survey data illustrates the potential flexibility of this instrument in both scaling up research on everyday life (Browne et al., 2013) and enhancing understandings of the couple relationship. It highlights the prevalence of everyday gestures in sustaining long-term relationships. Without it, we would have been unaware of the significant role of small thoughtful acts in symbolizing a partner’s generosity and attentiveness and the extent to which these feature in couples’ everyday interactions. It also made us attuned to the underlying biographical, structural and socio-cultural factors that so resoundingly shape couple relationships.

The deployment of moments provides a lens through which we can focus in on the minutiae of everyday experience, to gain insight on wider processes, meanings and cross-cutting analytical themes. Moments invoke a sense of the ephemeral, fleeting and transitory. However, like other examples in the *Enduring Love?* project dataset, the adventure scenario described by Sumaira is anything but momentary. Its ordinariness sets it apart from the epiphanal or fateful (Giddens, 1991) but its emotional vibrancy means it becomes an *enduring moment* that lives on and stays with the couple, becoming part of their relationship narrative. Similarly the emotional intensity of the moment in which Hayley resents being woken but worries about her selfishness reveals her ongoing struggle to negotiate the intimate dynamics of her relationship, both in the present and in an imagined future with her partner. In this, then, these everyday revelatory moments are significant – analytically and personally. They comprise emotional scenarios (Burkitt, 2014) through which the participant may gain greater personal insight and the researcher increased understanding. At the same time their vital intensity hails the reader, bringing them (you) into the relationship story and the analytical endeavour. For the project and its broader analytical aims, moments like those examined here stand as examples of the spatial–temporal–embodied relationship practices that couples do – *every day* – to sustain their relationships. What this approach can and does provide, then, is rich insight into the complexities of relationships whilst ensuring that feelings and emotionality remain firmly attached. Insights afforded may ultimately unsettle and undo certainties but, by remaining firmly attached to the value of the everyday for interrogating couple relationships, then perhaps we can be more attentive to the contested and ambivalent ways in which we live and love in these contemporary times.

## Notes

1. The qualitative sample (*n* = 50) comprised heterosexual and LGBT couples, aged 18–65 years. The online survey (*n* = 5445) comprised UK (*n* = 4494), US (*n* = 727), and RoW (*n* = 224) responses.

2. Coding was completed using NVivo 10 data management software.
